# Should neurosurgeons continue to work in the absence of personal protective equipment during the COVID-19 era?

**DOI:** 10.1007/s00701-021-04703-8

**Published:** 2021-01-20

**Authors:** Marleen Eijkholt, Alexander Hulsbergen, Ivo Muskens, Tiit Illimar Mathiesen, Ciaran Bolger, Zeev Feldman, Neil Kitchen, Nicolás Samprón, Ulrika Sandvik, Magnus Tisell, Marike Broekman

**Affiliations:** 1 Department of Medical Ethics and Health Law (Department of Neurology), Leiden University Medical Centre, Leiden, the Netherlands; 2 Department of Neurosurgery, Haaglanden Medical Center/Leiden University Medical Center, Leiden, the Netherlands; 3 Department of Clinical Medicine, Rigshospitalet - Neurocentret, Copenhagen, Denmark; 4 Department of Clinical Medicine, University of Copenhagen, Copenhagen, Denmark; 5 Department of Clinical Neuroscience, Section for Neurosurgery, Karolinska Institutet, Stockholm, Sweden; 6 Beaumont Hospital, Dublin, Ireland; 7 Pediatric Neurosurgery, Sheba Medical Center, Ramat Gan, Israel; 8 National Hospital Queen Square, London, England UK; 9 Servicio de Neurocirugía, Hospital Universitario Donostia, San Sebastian, Spain; 10 Department of Clinical Neurosciences, Karolinska Institutet, Solna, Sweden; 11 Department of Neurosurgery, Sahlgrenska University Hospital, Gothenborg, Sweden

**Keywords:** COVID-19, Neurosurgeons, Duty to work, Ethical, PPE

## Abstract

The COVID-19 pandemic has resulted in a widespread shortage of personal protective equipment (PPE). Many healthcare workers, including neurosurgeons, have expressed concern about how to safely and adequately perform their medical responsibilities in these challenging circumstances. One of these concerns revolves around the pressing question: should providers continue to work in the absence of adequate PPE? Although the first peak of the COVID-19 crisis seems to have subsided and supply of PPE has increased, concerns about insufficient PPE availability remain. Inconsistent supply, limited efficacy, and continued high demand for PPE, combined with the continued threat of a second COVID-19 wave, mean that the issues surrounding PPE availability remain unresolved, including a duty to work. This paper offers an ethical investigation of whether neurosurgeons should perform their professional responsibilities with limited availability of PPE. We evaluate ethical considerations and conflicting duties and thereby hope to facilitate providers in making a well-considered personal and moral decision about this challenging issue.

## Introduction

The coronavirus disease 2019 (COVID-19) crisis has resulted in a widespread shortage of adequate personal protective equipment (PPE). Many healthcare workers, including neurosurgeons, are not sure how to safely and adequately perform their medical responsibilities in these challenging circumstances. Some healthcare workers have even expressed concerns about going to work that stem from conflicts between their willingness to care for COVID-19 patients and the safety of themselves, their families, and non-COVID-19 patients [3, 5, 14, 13, 23, 25]. Indeed, COVID-19 incidence and mortality are considerably higher among healthcare professionals (HCPs) when compared with workers in other sectors, justifying these concerns [1, 6, 7, 29].

Although the first peak of the COVID-19 crisis may have passed in most developed countries and the issues around sufficient supply of PPE might have decreased, concerns about insufficient and inadequate PPE remain. Inconsistent supply, limited efficacy, and continued high demand for PPE exacerbate these concerns in light of the threat of a second COVID-19 wave due to loosening of social distancing restrictions. These hard questions will likely play a role in the short- to intermediate-term future and warrant an ethical evaluation of whether neurosurgeons should perform their professional duties with limited availability of PPE.

This article aims to perform such an investigation. We evaluate several ethical considerations and conflicting duties concerning work in the absence of adequate PPE. Our focus on these duties comes first and foremost from the health care provider’s role, which by its nature is defined by duties. At the same time, this focus highlights provider’s different roles and responsibilities, including duties toward different stakeholders that may become apparent during a pandemic. While a public health crisis like the present one is a unique scenario in which the focus shifts away from an everyday patient-centered focus to include other interests more explicitly, the duty-based framework is still preferable over other frameworks. Duties can exist *a priori* and are not dependent on outcomes, which may be uncertain especially in a pandemic. A duty-based framework can also account for various professional and non-professional relations rather than to focus on ultimate virtues in any particular relation. At the same time, the duty-based framework can account for neurosurgeons as individual moral agents with obligations and rights who must make personal decisions for themselves, their patients, and the community. With our overview, we hope to facilitate such decisions regarding these challenging issues.

## Limited legal and professional obligations

The decision about whether or not to go to work when facing shortages of PPE is a personal and moral assessment rather than a matter of legal and professional obligations. Contractual and non-contractual legal obligations have limited relevance in this context, and typically only apply when there is a safe neurosurgical work environment. Professional standards do not offer much guidance, even when they acknowledge that professionals might have to work in risky and even unsafe settings. The World Health Organization (WHO), for example, has explicitly acknowledged that “the duty to work notwithstanding risks to one’s health is not unlimited” [30]. The American Medical Association, the Canadian Medical Association, and some European societies have adopted similar positions [18]. Provisions from the early days of the previous century used to state that physicians should work “even at the jeopardy of their own lives”[8], but such firm obligations are no longer in place [27]. Medical professional guidelines refer to the autonomy of the physician and accede to other responsibilities and interests on the side of the professional even in times of a pandemic [4]. At risk of losing members or support, professional or other health organizations are also unlikely to install and enforce such duties upon their individual members at this point in time.

Consequently, the decision to work is a matter of personal appreciation that includes perceptions on risk and assessment of what risks are unreasonable, instead of a forceful imperative of a social contract between health care providers and the general public. Any duty to society, which is part of the social contract and which we will further describe below, seems to have become less definitive amidst the acknowledgment of other responsibilities of the professional. Clear-cut guidelines about the boundaries of the professional responsibilities are not available to the neurosurgical community, or physicians in general, when it comes to the unprecedented circumstances in the COVID-19 era and the potentially limited availability of adequate PPE.

## Risk assessment: which risks are acceptable?

Some have argued that the risks that come with care for COVID-19 patients are unacceptable when PPE is limited [26]. These unacceptable risks would allow physicians to decline work responsibilities as a result. Neurosurgical care for COVID-19 patients with limited PPE comes with a considerable personal risk of exposure when compared with non-medical professions. Neurosurgeons must get close to patients and perform invasive and potentially aerosol-generating procedures [19]. Yet, one could argue that neurosurgeons accept these risks when they take up a profession in health care and that they sign up for a moral duty to work even in times of elevated health risks. This interpretation supports providing care of COVID-19-positive patients when PPE is available. The severity of the COVID-19 pandemic does not discharge providers of this duty [28]. However, the absence of PPE increases the risk of contracting COVID-19 to a level that, arguably, falls outside the usual range of risk-exposure for neurosurgeons. Neurosurgeons might have never signed up to accept such a level of risk.

Hence, additional assessment is necessary to determine what risks are (un)reasonable. Risks could include medical, social, familial, or mental health risks for the neurosurgeon and their family members. Risks are the product of anticipated harms and their respective probabilities. The likelihood of contracting COVID as an HCP is estimated to range from 8 to 30% [10–12, 24]. The estimated probability of mortality for HCPs is 0.001% [20]. These numbers are just averages, and the risk of death for an elderly overweight doctor could be much higher. The acceptability of the calculated risks will mostly depend on personal judgment. If the risks of adverse outcomes in particular situations are exceedingly high, it may be necessary to protect HCPs regardless of their choice to provide care. A provider who accepts a certainty of death in their line of professional duty, either for themselves or for their family members, would have to be stopped for lack of professional judgment. Yet, beyond such extreme scenarios, no single or simple tool can determine what risks are acceptable if the basic safety equipment is missing. We recognize at least three determinants of the acceptability of risk: factors related to the likelihood of contracting COVID-19, factors associated with the severity of adverse outcomes, and factors related to a duty to work.

## Which factors determine the likelihood of contracting COVID-19?

The rapidly changing nature of the pandemic, the increased demands on healthcare systems, the varying availability of PPE, and the local variations in the density of infected patients complicate the estimation of the probability of contracting COVID-19. Uncertainty about acceptable viral loads further complicates this calculation. The effectiveness of PPE itself depends on its nature and its quality. For instance, there may be a shortage of N95 masks when suboptimal PPE (e.g., regular surgical masks) remains available. Suboptimal equipment may offer some protection and may be acceptable in some scenarios, but this level of protection will not provide enough safeguards in many cases. At the same time, PPE might not be entirely fitting for the individual providers, i.e., most equipment is based around male models and becomes uncomfortable when worn for extended periods [15].

A neurosurgeon’s probability of getting infected or suffering adverse outcomes is also determined by individual factors such as age, body mass index, or comorbidities. Moreover, neurosurgeons must consider the nature, duration, and frequency of neurosurgical procedures. Endoscopic endonasal surgery, for instance, will come with higher chances of exposure than procedures that do not generate aerosols or require ventilation [21]. The duration of potential exposure for neurosurgeons may further increase the risk of COVID-19 contraction, as many neurosurgical interventions entail lengthy assessments and operations.

## Conflicting duties

Besides the actual factors determining the probability of getting infected, neurosurgeons must weigh the implications of either contracting COVID-19 or the discontinuation of care to decide what risks are reasonable and acceptable. The importance of these implications and the complexity of this calculation are determined by a series of potentially conflicting duties that are summarized in Table 1 and outlined below. These duties illustrate that providers have different roles and responsibilities. Neurosurgeons, consequently, might be confronted with conflicting ethical duties, such as a duty to oneself versus a duty to the community. Modern society, however, ultimately places the decisions about how to weigh these duties on individual providers. Traditional perceptions about the health care providers’ elevated duties to the community have been reduced, due to diminished professional privileges and status, so that the individual provider is freer to exercise their individual rights and choices. This overview of duties can help structure such individual decision-making.

**Table 1:** Duty to work in the absence of PPE in the COVID-19 era: conflicting duties

Duty	Favors working without PPE	Favors declining to work without PPE
Duty to patients	○ Delivering emergency care ○ Maintaining access to neurosurgical care ○ Preventing backlog of untreated cases	○ Minimizing the probability of becoming a super-spreader ○ Avoiding patient fears of exposure resulting in delayed access to care and treatment
Duty to society	○ Continuing neurosurgical care as expected by society ○ Applying highly specific and essential skills of neurosurgeons	○ Maintaining high-quality standards of care, including safety ○ Acting upon the lack of reciprocity from society in the absence of PPE
Duty to family	○ Continuing work to sustain a family	○ Minimizing the risk of abandoning family due to death in the line of duty ○ Reducing the probability of infecting family members ○ Continuing care duties by the neurosurgeon towards their family members ○ Appreciating the irreplaceability of oneself in the family setting
Duty to colleagues	○ Spreading risks evenly if the risk profile is equal for all ○ Accepting burdens for colleagues at high risk and who need to be protected if the individual risk is relatively negligible ○ Accepting an individual’s place in the chain of workers with exceptional experience and skills	○ Getting infected may render someone unable to contribute to care for prolonged periods, which increases the long-term burden on colleagues ○ Promoting and securing that no one works without adequate protection
Duty to self	○ Respecting a neurosurgeon’s autonomous choice to continue care where possible ○ Avoiding moral distress	○ Jeopardizing personal health ○ Safeguarding irreplaceability of you as a person

### Duty to patients

Care for individual patients is the most immediate professional duty of any physician. The neurosurgeon has an obligation to the patient to perform adequate care. However, perceptions on this prima facie duty come with several concerns. The urgency of care, for example, might influence the interpretation of this duty. Immediate or short-term life-saving surgeries generate a more substantial responsibility than elective procedures. Concerns about a backlog of untreated cases might also impact providers’ interpretation of the duty, as this impacts patient access to future care. Denying care to patients is ethically contentious as patients are not responsible for the COVID-19 epidemic, contracting COVID-19, or the lack of PPE. From the standpoint of equality and fairness, all patients should have equal access to treatment, including those patients that suffer from COVID-19. Denying these patients essential access to neurosurgery will result in increased morbidity and mortality.

On the other hand, working without PPE entails other patient-related concerns that impact perceptions on duty to work. Neurosurgeons might become super-spreaders when they contract COVID-19 and harm many other patients [16]. Patients may also choose to avoid treatment by neurosurgeons who work without optimal PPE. Patients are already avoiding the healthcare system out of concern of getting infected in many countries. These concerns deprive patients of necessary neurosurgical care, which negatively impacts patients’ outcomes. After all, a physician’s duty to patients also extends to future patients.

### Duty to society

The duty to society at large supports the continuation of neurosurgery even during a pandemic. A lack of available PPE does not change this. Expectations around a continued duty to treat stem from a social contract between the society and the neurosurgical profession. Under this contract, physicians provide their expert services and dedication to individual patients and society in return for respect, status, autonomy in their practice, and substantial financial compensation [9]. The society hugely invests in the access to and quality of healthcare and also funds neurosurgical training. Neurosurgeons are both trusted and ethically obliged to provide continued high-quality care in return. The society may also expect that highly trained neurosurgeons, who possess limited and unique expertise, take their responsibility and use these skills to treat COVID-19 patients. This responsibility, for instance, applies to ruptured aneurysm clipping and neuro-intensive care management. Neurosurgeons rightly expect reciprocity in the form of first access to high-quality PPE. This exchange ensures the safest care for both patients and neurosurgeons and forms a moral force for a responsibility to work.

### Duty to family members

Neurosurgeons are also individuals with a family and social life, which gives rise to ensuing moral duties and potential conflicts. A moral conflict naturally arises when a neurosurgeon wants to provide care to patients but does not want to become a source of COVID-19 to family members. The WHO statement does not explicitly recognize the needs of family members of healthcare professionals, but this does not make these needs irrelevant. The safety of family members is vital to the safety and well-being of every neurosurgeon. Contacts between neurosurgeons and their relatives may be limited in line with social distancing measures to limit potential COVID-19 transmission. However, it is unreasonable to ask healthcare workers to completely distance themselves from their closest relatives for extended periods (i.e., complete self-quarantine outside of work).

Family members are not part of the social contract outlined above and did not consent to indirect COVID-19 exposure. Moreover, children or older adults may not fully understand the situation. Family members may much depend on their daily care and may simply greatly care about their exposed relative. Family members themselves may be at higher risk of adverse outcomes when exposed to COVID-19 due to, e.g., obesity, age, or past medical history. On the other hand, family members may also depend on the income provided by the neurosurgeon for their daily lives. Over the long term, neurosurgeons whose income is surgical volume-dependent may suffer financial detriment from an inability to work. Thus, the ability to financially sustain household members could be taken into consideration as part of the primary duty not to sacrifice oneself in the line of professional responsibilities.

### Duty to colleagues

Neurosurgery often requires a team of professionals that work around the clock. The duty to be a good colleague includes sharing the workload and the hazards of the workplace. If a provider decides not to consent to work or to show up, they increase the burden of care on other neurosurgeons and forsake a duty to spread the workload [17, 22]. The severity of this problem increases when the number of healthy providers becomes limited. Conversely, a surgeon that contracts COVID-19 may become unable to contribute to patient care for extended periods and, consequently, increase the workload for colleagues.

The (ir)replaceability of neurosurgeons severely complicates the question surrounding a duty to work. Neurosurgical training is lengthy and expensive. The number of neurosurgeons is also limited in most countries. This lack of neurosurgeons is even more relevant for specific subspecialties of neurosurgery that require long training periods, such as for neurovascular and skull base surgery. Experienced neurosurgeons that can perform such procedures may be of relatively advanced age and have a higher risk of adverse outcomes if they contract COVID-19. The loss of even a single specialized surgeon could endanger access to adequate care or require patients to travel long distances. Neurosurgeons should not discharge the duty of fairness to colleagues by shifting the duty to work to healthy young neurosurgeons who also face adverse outcomes.

### Duty to oneself

Obligations that come with the neurosurgical profession do not include a requirement to jeopardize personal health. The Geneva Declaration, for instance, explicitly states: “I will attend to my own health, well-being, and abilities in order to provide care of the highest standard” [31] which is further confirmed by the WMA statement on epidemics and pandemics [32]. Some healthcare providers may be very susceptible to COVID-19 or possibly experience adverse outcomes due to past medical history, age, or sex. Other neurosurgeons may feel a vocation to provide care to severely suffering patients, whatever the circumstances. Indeed, most people sign up for the medical profession to relieve suffering and care for patients. Not being able to do so could result in moral suffering.

Accordingly, the willingness of a neurosurgeon to provide healthcare without adequate PPE, including their willingness to accept high risks, is a critical factor in the decision to work. Such a decision remains within the autonomy of the neurosurgeon, as long as the dangers to others are limited.

## Collaboration can empower ethical decision-making

The individual autonomy of a neurosurgeon is a central component in the decision-making progress about providing care to COVID-19 patients when PPE is limited. Still, it may not be ideal to leave considerations entirely up to individuals without any external support. Some neurosurgeons may be willing to accept higher risks of contracting COVID-19 compared with more risk-averse colleagues. This unequal spread of chances to contract COVID-19 may be further complicated if those exposed are also those with higher probabilities of adverse outcomes. Risks of contracting COVID-19 would seem to increase with repeated exposure [2]. Unequal sharing of risks may negatively impact long-term availability and continuation of neurosurgical care.

Therefore, parties involved in neurosurgical care need to elaborate on what constitutes the duty to work, which risks are acceptable, and how to solve the questions discussed in this article. This elaboration could happen on a local, national, and international level. Striking a balance and coming to ethically acceptable decisions depends strongly on the local access to PPE, local neurosurgical healthcare structure, and the current local severity of the COVID-19 pandemic. Neurosurgical societies could provide reports and guidelines to help institutions continue care in their local healthcare system ethically. All parties involved should also look outside of neurosurgery for innovative solutions but keep the uniqueness of neurosurgery in mind. Continuous elaboration may result in a broad consensus on what risks are acceptable. This consensus needs constant updating by all parties involved as the COVID-19 pandemic progresses. Consensus may empower the neurosurgical community to make ethical decisions and ensure continuity of quality health care. In the end, both neurosurgeons and patients will benefit from an optimal continuation of high-quality care in the long run.

## Conclusion

The decision to work in the absence of PPE in the COVID-19 era is a personal one that involves weighting factors like the probability of contracting COVID, the urgency and nature of the neurosurgical procedure, as well as conflicting duties to family members, society, and the neurosurgeon as a person. No legal or professional guidelines adequately prescribe what the provider ought to do in the absence of PPE. Neurosurgeons, therefore, need to decide for themselves whether they deem the risks unreasonable and when to discontinue care for neurosurgical patients. Well-written guidelines can facilitate these decisions. Transparent data of individual risk and understanding of ethical principles are fundamental to make these well-informed and personal decisions. There is no duty to treat patients at the risk of one’s own life. Physicians may expect reciprocity when practicing their profession. Reflection and elaboration within the neurosurgical community will be essential to establish a spectrum of acceptable risks. A continued discussion may also result in a higher level of agreement about the reciprocity that neurosurgeons may expect. Such collaboration will hopefully ensure the continuation of high-quality neurosurgical care in the long run. At the same time, neurosurgeons must continue to balance duty to work and other duties in the absence of PPE.
